# Genetic diversity and population structure of *Plasmodium falciparum *in the Philippines

**DOI:** 10.1186/1475-2875-8-96

**Published:** 2009-05-08

**Authors:** Moritoshi Iwagami, Pilarita T Rivera, Elena A Villacorte, Aleyla D Escueta, Toshimitsu Hatabu, Shin-ichiro Kawazu, Toshiyuki Hayakawa, Kazuyuki Tanabe, Shigeyuki Kano

**Affiliations:** 1 Department of Appropriate Technology Development and Transfer, Research Institute, International Medical Center of Japan, 1-21-1 Toyama, Shinjuku, Tokyo 162-8665, Japan; 2 Department of Parasitology, College of Public Health, University of the Philippines Manila, 625 Pedro Gil street Ermita, Manila, the Philippines; 3 Gunma University School of Health Sciences, 3-39-15 Showa-machi, Maebashi 371-8514, Japan; 4 National Research Center for Protozoan Diseases, Obihiro University of Agriculture and Veterinary Medicine, Inada 2-13, Obihiro, Hokkaido 080-8555, Japan; 5 Laboratory of Malariology, Research Institute for Microbial Diseases, Osaka University, Suita, Osaka 565-0871, Japan

## Abstract

**Background:**

In the Philippines, malaria morbidity and mortality have decreased since the 1990s by effective malaria control. Several epidemiological surveys have been performed in the country, but the characteristics of the *Plasmodium falciparum *populations are not yet fully understood. In this study, the genetic structure of *P. falciparum *populations in the Philippines was examined.

**Methods:**

Population genetic analyses based on polymorphisms of 10 microsatellite loci of the parasite were conducted on 92 isolates from three provinces (Kalinga, Palawan, and Davao del Norte) with different malaria endemicity.

**Results:**

The levels of genetic diversity and the effective population sizes of *P. falciparum *in the Philippines were similar to those reported in the mainland of Southeast Asia or South America. In the low malaria transmission area (Kalinga), there was a low level of genetic diversity and a strong linkage disequilibrium (LD) when the single-clone haplotype (SCH) was used in the multilocus LD analysis, while in the high malaria transmission areas (Palawan and Davao del Norte), there was a high level of genetic diversity and a weak LD when SCH was used in the multilocus LD analysis. On the other hand, when the unique haplotypes were used in the multilocus LD analysis, no significant LD was observed in the Kalinga and the Palawan populations. The Kalinga and the Palawan populations were, therefore, estimated to have an epidemic population structure. The three populations were moderately differentiated from each other.

**Conclusion:**

In each area, the level of genetic diversity correlates with the local malaria endemicity. These findings confirm that population genetic analyses using microsatellite loci are a useful tool for evaluating malaria endemicity.

## Background

Malaria is still one of the major public health problems in the Philippines, although the morbidity and the mortality have decreased in the last decade [1,2]. The Philippine archipelago comprises 7,109 islands in the western Pacific Ocean. Out of the 79 provinces, malaria is endemic to 65 provinces [3]. Epidemiological data suggest that malaria endemicity across the Philippines is quite different from one area to another. Therefore, it is likely that the parasite populations in each endemic area differ genetically.

Understanding the genetic structure of malaria parasites is essential to predict how fast phenotypes of interest, such as novel antigenic variants or drug resistance, originate and spread in populations [4,5]. Anderson *et al *reported a spectrum of population structures in *Plasmodium falciparum*: in high transmission areas, such as sub-Saharan Africa, weak linkage disequilibrium, high genetic diversity and low levels of genetic differentiation between populations were observed, while in low transmission areas, such as the Brazilian Amazon, significant linkage disequilibrium, low genetic diversity and high levels of genetic differentiation between populations were observed [6].

Population genetic analyses of *P. falciparum *in Southeast Asia have been reported from Thailand and Malaysian Borneo [6,7]. In the Philippines, several epidemiological surveys and some molecular biological studies on drug resistant malaria and antigenic molecules have been reported [1,2,8-11]. However, basic genetic analyses of the parasite populations in the country using multilocus neutral markers have not been reported so far.

The objective of the present study was to estimate the genetic structure of *P. falciparum *populations in the Philippines using 10 highly polymorphic microsatellite loci and to discuss the correlation between the levels of genetic diversity and the level of malaria endemicity in this country. To investigate *P. falciparum *populations in the Philippines, the population genetic analyses were conducted using a method consistent with those used to assess the genetic structure of the parasite populations in other countries, so as to enable a comparison of the present data with others [6,7,12]. The authors also considered the implications for the emergence of anti-malarial drug resistance, based on the data of this study and data of the other populations that were previously reported from other countries [6,7,12].

## Methods

### Collection sites and epidemiological data

**Figure 1:**
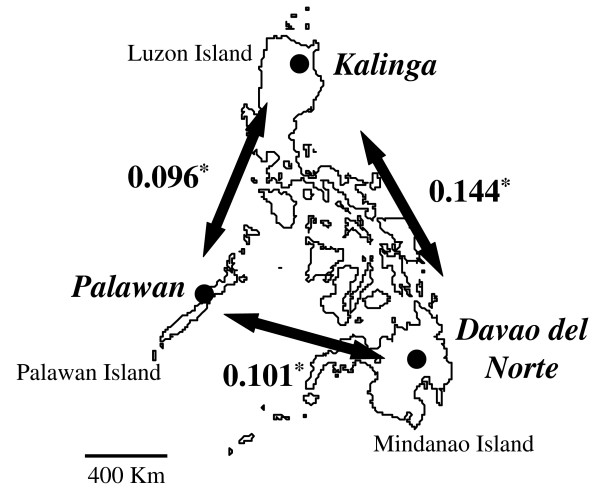
**Study areas in the Philippines**. Levels of genetic differentiation (*F*_ST _values) for each pairwise population comparison are shown with numbers and arrows. All values are significantly different from 0 (* *P *< 0.001).

The study areas, sample sizes (n), and years of the patient blood collection were as follows: Kalinga province (Northern part of Luzon Island), n = 22, collected by field surveys in 2003 through 2005, Palawan province (Palawan Island), n = 40, collected by field surveys in 2003 through 2006, and at Davao Regional Hospital in Tagum City, Davao del Norte (Southeastern part of Mindanao Island), n = 30, collected in 1999 through 2001 (Figure 1). These three study areas are sentinel provinces in the Philippines, where malaria is endemic perennially, but do not represent all the *P. falciparum *endemic foci of the country. The field isolates were collected in Tabuk, Kalinga province, where slide positivity rate (SPR) and annual parasite incidence (API; per 1,000 people/year) were 4.3 and 3.6 respectively (2004). Other isolates were collected in Puerto Princesa City, Palawan province where SPR and API were 18.5 and 16.3 respectively (2004). The other isolates were collected in Davao del Norte province, which had a 4.5–15.2, SPR and 2.3–7.8, API (1999–2001). APIs were collected from Annual Reports of the Provincial Health Offices of Kalinga, Palawan and Davao del Norte. Based on this epidemiological information, Kalinga can be classified as hypoendemic, Palawan as mesoendemic, and Davao del Norte as hypoendemic to mesoendemic. Information pertaining to entomological inoculation rates was not available to this study.

Informed consent was obtained from all the patients prior to the blood collection. This study was given ethical approval by the University of the Philippines, Manila, and performed according to the ethical guidelines for epidemiological studies provided by the Ministry of Education, Culture, Sports, Science and Technology and the Ministry of Health, Labour and Welfare of Japan.

### DNA extraction, PCR amplification

The patient blood samples were frozen in liquid nitrogen or preserved on filter papers (IsoCode Stix, Schleicher & Schuell co., Germany) and kept at room temperature until examined. The parasite DNA was extracted from frozen whole blood samples by phenol-chloroform extraction after proteinase K digestion or from the blood-spot samples on the filter papers, as per manufacturer's instructions [13].

### Genotyping

Ten microsatellite DNA loci were amplified by semi-nested PCR. The loci were as follows: TA1 (chromosome 6), TA40 (chromosome 10), Poly α (chromosome 4), Pfg377 (chromosome 12), PfPK2 (chromosome 12), TA109 (chromosome 6), TA87 (chromosome 6), TA81 (chromosome 5), TA42 (chromosome 5) and 2490 (chromosome 10). The PCR primer sets and amplification conditions were consistent with the protocol of Anderson *et al *using a modified TA40 primer set [14,15]. Sizes of fluorescence-labelled PCR products were measured on an Applied Biosystems Prism Genetic Analyzer 310 using Gene Scan version 3.1.2 with a 500 ROX size standard (ABI, CA, USA).

Different-sized PCR products amplified using the same primer set were considered to be individual alleles within a locus, as size variation among isolates is consistent with the repeat number in a microsatellite locus [14]. The electropherogram shows peak profiles for the microsatellite loci, based on fluorescence intensity of the labelled PCR products in this analysis. Multiple alleles per locus were scored if minor peaks were taller than at least one-third the height of the predominant allele for each locus. Multiple-genotype infections (MGIs) were defined as those in which at least one of the 10 loci contained more than one allele [6].

### Data analysis

Expected heterozygosity (*H*) was calculated for each locus based on the allele frequencies of the 10 examined microsatellite loci. *H *values were calculated using *H *= [n/(n - 1)] [1 - ∑*pi*^2^], where n corresponds to the number of isolates examined and *pi *is the frequency of the *i*th allele.

Effective population size (*Ne*) was estimated based on *H *and the microsatellite mutation rate (*μ *= 1.59 × 10^-4^; 95% confidence interval: 6.98 × 10^-5^, 3.7 × 10^-4^) for *P. falciparum *[16-18]. The infinite-alleles model (IAM) and stepwise mutation model (SMM) were used to estimate *Ne*. For the IAM, the formula *Neμ *= *H*/4(1 - *H*) was used, whereas for the SMM, the formula *Neμ *= 1/8{[1/(1 - *H*)]^2 ^- 1} was used [17,18].

Each population was examined for evidence of a recent genetic bottleneck (ie, a severe decrease in population size). Heterozygosity excess was used for evidence of genetic bottlenecks [19,20]. In a population that underwent a bottleneck, heterozygosity excess is observed, in which the value of *He *(the observed Hardy-Weinberg equilibrium heterozygosity) is greater than that of *H *(expected heterozygosity based on the number of alleles and the sample size). The mode shift in allele frequency distribution for the presence of rare alleles was also examined. A normal L-shaped distribution indicates evidence of a non-bottlenecked population, whereas a shifted mode of distribution indicates evidence of a bottlenecked population. The BOTTLENECK program, version 1.2.02, was used to search for evidence of heterozygosity excess and mode-shift [19,21]. The Sign Test and the Wilcoxon Signed-Rank Test were used to evaluate statistical significance.

Multilocus linkage disequilibrium was assessed using the standardized index of association (*I*_A_^S^) [22,23]. This analysis was performed using the LIAN 3.5 Web interface [24]. *I*_A_^S ^was calculated using the formula *I*_A_^S ^= (*V*_D_/*V*_e _- 1)/(*l *- 1) with permutation testing of the null hypothesis of complete linkage equilibrium (*I*_A_^S ^= 0), where *V*_D _is the observed mismatch variance, *V*_e _is the expected mismatch variance, and *l *is the number of examined loci. Significances of the observed *I*_A_^S ^values were calculated by Monte-Carlo simulation, using 10,000 random permutations of the data. This statistic is a variation of the method proposed by Maynard-Smith *et al *[22]. The results were standardized by the number of loci, to enable a comparison of different data sets [6,22]. This test was applied to the data sets from each population in two ways. First, the mixed-clone infections were excluded so that only the single-clone infections were analysed, giving absolute confidence in the haplotype profile. Second, any multilocus genotype found in more than one isolate was only counted once in the analysis, ie unique haplotypes only, reducing the sample size slightly and thereby removing the possible effect of recent epidemic expansion of particular clones [6]. Since the LIAN cannot analyse isolates possessing missing data, the 57 isolates obtaining the allelic data of 10 loci were used.

The extent of population subdivision between the populations in the Philippines was estimated using Weir and Cockerham's Ø estimator for determining F statistics (*F*_ST_) [25]. *F*_ST _were calculated using the FSTAT program version 2.9.3.2 and tested for significant difference from 0, based on 1,000 random permutations of the data set [26].

## Results

Allele frequencies of each locus in the three populations are shown in Additional File 1. Of the total of 92 isolates examined, allelic data of the microsatellite loci were obtained from the 74 isolates (80.4%)(16 from Kalinga, 28 from Palawan and 30 from Davao del Norte), while in the remaining 18 isolates (19.6%), the allelic data were not available (6 from Kalinga and 12 from Palawan) because of failures in acquiring PCR products during the genotyping. Multiple-genotype infection (MGI) was observed in some loci in three isolates from the Palawan population, and two isolates from the Davao del Norte population. No MGI was observed from the Kalinga population. Data from loci that originated from MGI were excluded from the analyses.

### Genetic diversity

The genetic diversity of each population was assessed by determining the number of alleles per locus in each population and by calculating the expected heterozygosity (*H*) (Table 1). The mean numbers of alleles ± SE of the Kalinga, Palawan and Davao del Norte populations were 2.40 ± 0.37, 4.80 ± 0.83 and 4.50 ± 0.87, respectively. The mean of *H *± SE of the Kalinga, Palawan and Davao del Norte populations were 0.39 ± 0.10, 0.60 ± 0.09 and 0.51 ± 0.08, respectively.

**Table 1:** Mean numbers of alleles and expected heterozygosity (*H*) in the three *P. falciparum *populations

**Population**	**No. of allele mean ± SE**	**Expected heterozygosity (*H*) mean ± SE**
Kalinga	2.40 ± 0.37	0.39 ± 0.10

Palawan	4.80 ± 0.83	0.60 ± 0.09

Davao del Norte	4.50 ± 0.87	0.51 ± 0.08

### Effective population size

*Ne *values of the three populations were calculated from the mean expected heterozygosity and mutation rates of *P. falciparum *microsatellite loci using the IAM and the SMM (Table 2). The sizes of the Kalinga, Palawan and Davao del Norte populations were 997, 2388 and 1650, respectively, based on the IAM, and 1313, 4202 and 2515 respectively, based on the SMM.

**Table 2:** Effective population sizes (*Ne*) of the three *P. falciparum *populations

**Population**	**IAM**	**SMM**
Kalinga	997 (428, 2,271)	1,313 (564, 2,991)

Palawan	2,388 (1,026, 5,440)	4,202 (1,806, 9,572)

Davao del Norte	1,650 (709, 3,758)	2,515 (1,081, 5,729)

### Genetic bottleneck

Evidence of genetic bottlenecks was assessed based on heterozygosity (*H*) excess and patterns of allele frequency distribution (ie, mode-shift) [19,20]. Table 3 shows the number of loci that correspond with *H *excess and deficiency. Statistically high levels of *H *excess compared with *H *deficiency (*P *< 0.05) were observed in the Kalinga and the Palawan populations when the IAM was applied to the analyses, indicating evidence of genetic bottleneck events. In contrast, there was no significant difference between the levels of *H *excess and *H *deficiency in the Davao del Norte population when the IAM and the SMM were applied to the analyses, indicating the absence of a genetic bottleneck event.

**Table 3:** Observed versus expected heterozygosity in the three *P. falciparum *populations

Population	No. of loci	IAM			SMM			Mode-shift
			
		*H* excess	*H* deficiency	*P*	*H* excess	*H* deficiency	*P*	
Kalinga	7	6	1	*P *< 0.05	5	2	NS	NA

Palawan	9	8	1	*P *< 0.05	3	6	NS	NA

Davao del Norte	10	5	5	NS	4	6	NS	Normal

Proportion of loci with excess or deficiency in observed heterozygosities, relative to expected heterozygosities. Data were subject to mutation drift equilibrium via IAM and SMM, as well as mode-shift analyses. *H*: heterozygosity. NS: not significant. NA: not applicable. a: sign test, b: Wilcoxon sign-rank test, normal: normal L-shaped distribution = nonbottlenecked population, shifted: shifted mode = bottlenecked population.

In the Davao del Norte population, the mode-shift indicator test showed a normal L-shaped distribution, indicating evidence of a non-bottlenecked population. This test could not be applied to the other two populations due to small sample sizes (<30) [21].

### Multilocus linkage disequilibrium

*I*_A_^S ^values were calculated for the three populations, with permutation testing of the null hypothesis of *I*_A_^S ^= 0 (equilibrium of multilocus frequencies) (Table 4). *I*_A_^S ^values of the three populations were highly variable. When the single-clone haplotype was used in the analysis, the *I*_A_^S ^values ranged from 0.043 to 0.104, whereas when the unique haplotypes were used in the analysis, the *I*_A_^S ^values ranged from 0.003 to 0.029. Significant linkage disequilibrium was observed in all three populations (*P *< 0.05) when the single-clone haplotype was examined using the 10 loci in the analyses. However, no significant linkage disequilibrium was found in the Kalinga and Palawan populations when the unique haplotypes were examined in the analyses.

**Table 4:** Multilocus linkage disequilibrium in the three *P. falciparum *Populations

Population	Single Clones		Unique Haplotypes Only	
	
	No.	*I*_A_^S^	No.	*I*_A_^S^
Kalinga	13	0.104***	7	0.003

Palawan	16	0.043**	14	0.011

Davao del Norte	28	0.052**	24	0.029*

Single clones show all haplotypes in single-clone infections. Unique haplotypes show haplotypes excluding duplicates of any multiply represented infection. No. indicates the number of isolates for each measure. *P < 0.05, ** *P *< 0.01, *** *P *< 0.001

### Genetic differentiation

Levels of genetic differentiation between each pair of the three different populations were indicated by *F*_ST _values using the Weir and Cockerham estimator [25,26]. The values for each of the pairwise population comparisons ranged from 0.096 to 0.144. All values were significantly different from 0 (*P *< 0.001) (Figure 1).

## Discussion

The failure to obtain allelic data of the 18 isolates might be due to low parasite density and/or misdiagnosis of other malaria species such as *P. vivax *by microscopic observations in the field during the surveys in Kalinga and Palawan. The samples of Davao del Norte were collected at a hospital where more accurate microscopic diagnosis was feasible. A possibility that differences in sample size between the Kalinga population and the other two populations might have affected the results of the analyses cannot be ruled out, and this could be the limitation of the study. However, some of the data analyses such as expected heterozygosity (*H*), effective population size (*Ne*) and multilocus linkage disequilibrium (*I*_A_^S^), were corrected for sample size, which enabled an appropriate comparison of different data sets.

Levels of genetic diversity of the three *P. falciparum *populations in the Philippines were different from each other. The Kalinga population showed a relatively low number of alleles and low levels of expected heterozygosity (*H*), being similar to those of South American populations of *P. falciparum *(*H*: 0.30–0.40) [6,12]. In contrast, the Palawan and the Davao del Norte populations showed intermediate levels of *H*, being similar to those of mainland Southeast Asian (*H*: 0.51), Papua New Guinean (0.62–0.65), and Malaysian Borneo (0.46–0.63) populations of *P. falciparum *[6,7].

The levels of genetic diversity seemed to be correlated with the levels of malaria endemicity. The API in Kalinga province was lower than that of the other 2 provinces. According to the report from the Center for Health Development-CAR in Kalinga province, the mean of API ± SD was 4.8 ± 1.9 (1996–2005). According to the report from the Kilusan Ligtas Malaria in Palawan province, the mean of API ± SD was 15.6 ± 4.4 (1999–2005). This difference of malaria endemicity between the two provinces probably affected the levels of genetic diversity of the two populations of *P. falciparum*.

Levels of genetic differentiations (*F*_ST_) between the populations of *P. falciparum *in the Philippines (0.096–0.144) were moderate, and were similar to those between the mainland Southeast Asian and Papua New Guinean populations of *P. falciparum *(0.121–0.140), and lower than those between Sabah and Sarawak populations of *P. falciparum *(0.217–376) in Malaysian Borneo, as previously reported [6,7].

In the present study, possibilities of genetic bottlenecks with the Kalinga and the Palawan populations of *P. falciparum *were found. However, the significant heterozygosity excess (evidence of genetic bottlenecks) was observed only when the IAM was used for the analysis. This result is not robust because modes of microsatellite mutation theoretically fit the SMM. Luikart and Cornuet recommended that only the SMM should be used for microsatellite data to test for genetic bottlenecks, because the IAM may detect heterozygosity excess even in non-bottlenecked populations [20].

Significant multilocus linkage disequilibrium was obtained from all three populations of *P. falciparum *in the Philippines, when a single clone was used in the analysis [22-24]. The Kalinga and the Palawan populations showed decreased linkage disequilibrium with no levels of significance when the unique haplotypes were included in the data set. This is because the identical haplotype was frequently found from two or more isolates (patients) within the same population in Kalinga and Palawan. In particular, in the Kalinga population, 57% of the haplotypes (four out of seven haplotypes) were shared among two or more isolates (patients). In fact, such isolates were collected from the same site and on the same day. Maynard-Smith *et al *proposed simple methods to distinguish between "clonal" population structure and "epidemic" population structure of microbial pathogens [22]. They demonstrated that epidemic population structure could be identified by treating the multiply-represented haplotype as single individuals and then re-examining the linkage disequilibrium. If a decreased level of linkage disequilibrium is observed in the second analysis, the population is estimated to have an epidemic population structure. The results indicated that the Kalinga and Palawan populations had an epidemic population structure.

On the contrary, the Davao del Norte population persisted with significant linkage disequilibrium (*P *< 0.05) even when the multiply-represented haplotype was treated as single individuals. This result indicated that a recombination rate was estimated as low in the Davao del Norte population, and that the population has possibly not experienced the recent epidemic expansion of a particular haplotype.

Generally, levels of malaria transmission have a negative correlation with levels of linkage disequilibrium [6,7,12]. In areas where malaria transmission is high, levels of linkage disequilibrium are likely to decrease because one malaria patient can be infected with more than two genetically different clones of the parasite, and a mosquito can frequently acquire more than two genetically different clones in the same blood meal. Then, meiotic recombination of the parasite genomes will occur in the mosquito midgut and decrease the levels of linkage disequilibrium. In contrast, in areas where malaria transmission is low, levels of linkage disequilibrium are likely to increase because, generally, the frequency of multiple-genotype infection (MGI) and the number of clones per patient are lower than those in high transmission areas, and the chances of acquiring more than two genetically different clones with one blood feeding by a mosquito will also be lower than those in high transmission areas.

In the present study, MGI in a patient was observed in the Palawan (three of 28 isolates: 10.7%) and the Davao del Norte (two of 30 isolates: 6.7%) populations, but not in the Kalinga population. MGI is important to the maintenance of genetic diversity of the *P. falciparum *population. Without multiple infections, this parasite has no chance of encountering genetically different clones in a mosquito midgut. In the present study, a clear correlation between the levels of genetic diversity (*H*) and the frequencies of MGI was found. The *H *values of the three populations were elevated as follows: Kalinga (0.39) < Davao del Norte (0.51) < Palawan (0.60), and the frequencies of MGI were also elevated as follows: Kalinga (0.0%) < Davao del Norte (6.7%) < Palawan (10.7%). In contrast, a negative correlation between the levels of linkage disequilibrium and the frequencies of MGI of the parasite was found. When single clones were used in the data set for the linkage disequilibrium analysis (Table 4), the levels of linkage disequilibrium were elevated as follows: Palawan < Davao del Norte < Kalinga, while the frequencies of MGI were elevated as follows: Kalinga (0.0%) < Davao del Norte (6.7%) < Palawan (10.7%). These results are consistent with the previously reported population genetics trends of *P. falciparum *[6,7,12].

However, caution is needed for detecting MGI by microsatellite typing, because it is likely to underestimate the prevalence of MGI of *P. falciparum*. In Brazil, over 40% of local isolates were MGI detected by a single-copy antigen-coding gene (*merozoite surface protein-1*: *msp-1*), however less than 20% of local isolates were MGI detected by microsatellite typing [6,27]. In fact, nested PCR genotyping of block 2 of the *msp-1 *gene and block 3 of *msp-2 *[28] in the present samples showed that more than 20% of the isolates of each of the three populations proved to be MGI (Additional File 2).

The levels of genetic differentiation between the three populations in the Philippines were moderate, and lower than those reported in Malaysian Borneo where the parasite populations were highly fragmented from each other [7]. High levels of genetic differentiation of the populations in Malaysian Borneo are considered to be a result of fragmentation of the population structure owing to an effective malaria control and low migration rate of people in the endemic areas. Therefore it is expected that an elevation of *F*_ST _values between the three populations in the Philippines will be detected in the near future, with the onset of effective malaria control.

However, the migration rate of people to the endemic areas in the Philippines may be different from that in Malaysian Borneo. In Malaysian Borneo, malaria endemic areas are typically in remote, isolated mountainous villages. In contrast, in the Philippines, malaria is not only endemic in remote areas (e.g. forests, mountainous villages) but also in relatively big cities or ports (e.g. Puerto Princesa City, Palawan province). For example, in Palawan, malaria is highly endemic in villages near seashores where fishermen live, and they sail from island to island, freely catching and selling fish.

Although mechanisms for the emergence of drug resistance have not been fully understood, levels of genetic diversity and characteristics of population structure of *P. falciparum *would be the key factors to estimate a potential of the emergence of drug resistance. Ariey *et al *demonstrated that a metapopulation concept would be applicable to explain the emergence of drug resistant malaria [29,30]. They showed that the *P. falciparum *populations in Southeast Asia and South America, where chloroquine resistance emerged, were consistent with a typical metapopulation structure, ie, parasite populations were differentiated in several subpopulations but not completely isolated from each other. The levels of genetic diversity and the characteristics of population structure of *P. falciparum *in the Philippines were similar to those reported in mainland Southeast Asia or South America where chloroquine resistant malaria emerged [6]. Indeed, Chen *et al *indicated that some chloroquine resistant mutations seemed to have emerged in the Philippines [10]. Moreover, a chloroquine resistant malaria determined by an in vitro chloroquine susceptibility test has been reported from Kalinga, Palawan and Davao del Norte [8,9,31]. The results in this study suggest that the populations in the Philippines have the potential to make the drug-resistant mutation(s) spread into different endemic areas in this country.

## Conclusion

The genetic diversity and population structure of *P. falciparum *in the Philippines were examined using 10 highly polymorphic microsatellite loci of the parasite. The levels of the genetic diversity and the frequencies of MGI correlated with the local malaria endemicity. On the other hand, the levels of linkage disequilibrium correlated negatively with the local malaria endemicity. Genetic population analyses of *P. falciparum *using the microsatellite loci provide insight not only into understanding the basic biology of this organism, but also the epidemiology and control of the disease.

## Competing interests

The authors declare that they have no competing interests.

## Authors' contributions

MI carried out the molecular genetic studies, performed the population genetic analyses and drafted the manuscript. PTR, EAV, ADE, THat and SIK collected the patients' blood samples as well as helping with the writing of the manuscript. THay and KT helped with the population genetic analyses and helped with the writing of the manuscript. SK participated in the design of the study, acquisition of funding, coordination and writing of the manuscript.

## Supplementary Material

Additional File 1 **Allele frequencies and number of isolates (n) of 10 microsatellite loci of the three *Plasmodium falciparum *populations in the Philippines**. Allele names refer to the base pair sizes of the product of polymerase chain reaction. Click here for file

Additional File 2 **Percentages of multiple-genotype infection (MGI) of *msp1 and msp2 *of the three *Plasmodium falciparum *populations in the Philippines**. No. indicates the number of isolates for each measure.*msp1*: block 2 of merozoite surface protein-1 gene, msp2: block 3 of merozoite surface protein-2 gene, *msp1 *or *msp2 *represents the percentage of isolates that showed MGI in either *msp1 *or *msp2*. Click here for file
